# Advancing Personalized Strategies for Obesity Prevention Through Biologically and Behaviorally Tailored Interventions: The Strategic Framework for the BETTER4U Initiative

**DOI:** 10.1111/obr.70083

**Published:** 2026-02-22

**Authors:** Maria Kafyra, Ioanna Panagiota Kalafati, Susanne Bruins, Chrysa Episkopou, Delfien Gryspeerdt, Lisa Heggie, Saskia Kaltenbrunner, Stephan Kampshoff, Eva Karaglani, Hande Özkayagan Prändl, Ioannis Papathanail, Panagiotis G. Symianakis, Darya Silchenko, Vasiliki Vavouraki, Dorret Boomsma, Anastasios Delopoulos, Christos Diou, Anders Eriksson, Jaakko Kaprio, Paulina Krzywicka, Aleksandra Luszczynska, Yannis Manios, Eirini Marouli, Panagiotis Moulos, Stavroula Mougiakakou, René Pool, Karri Silventoinen, Nick Verhaege, Ruben Willems, George V. Dedoussis, the BETTER4U consortium

**Affiliations:** ^1^ Department of Nutrition and Dietetics, School of Health Science and Education Harokopio University of Athens Athens Greece; ^2^ Department of Nutrition and Dietetics, School of Physical Education, Sport Science and Dietetics University of Thessaly Trikala Greece; ^3^ Department of Biological Psychology Vrije Universiteit Amsterdam Amsterdam Netherlands; ^4^ Department of Electrical and Computer Engineering Aristotle University of Thessaloniki Thessaloniki Greece; ^5^ Department of Public Health and Primary Care Interuniversity Centre for Health Economics Research (I‐CHER), Ghent University Ghent Belgium; ^6^ The European Association for the Study of Obesity Dublin Ireland; ^7^ Department of Innovation and Digitalisation in Law University of Vienna Vienna Austria; ^8^ Science Communication Department European Food Information Council (EUFIC) Brussels Belgium; ^9^ ARTORG Center for Biomedical Engineering Research/AI in Health and Nutrition University of Bern Bern Switzerland; ^10^ Genome Analysis Athens Greece; ^11^ Department of Informatics and Telematics, School of Digital Technology Harokopio University of Athens Athens Greece; ^12^ Institute of Genomics University of Tartu Tartu Estonia; ^13^ Institute for Molecular Medicine Finland FIMM, HiLIFE University of Helsinki Helsinki Finland; ^14^ Institute of Psychology, CARE‐BEH Center for Applied Research on Health Behavior and Health SWPS University Wroclaw Poland; ^15^ European Centre for Obesity Harokopio University Athens Greece; ^16^ Institute of Agri‐Food and Life Sciences Hellenic Mediterranean University Research Centre Heraklion Greece; ^17^ William Harvey Research Institute, Barts and the London School of Medicine and Dentistry Queen Mary University of London London UK; ^18^ Institute for Fundamental Biomedical Research Biomedical Sciences Research Center 'Alexander Fleming' Vari Greece; ^19^ Population Research Unit, Faculty of Social Sciences University of Helsinki Helsinki Finland

**Keywords:** artificial intelligence, obesity, personalized lifestyle intervention, weight gain

## Abstract

The BETTER4U project (Preventing Obesity through Biologically and bEhaviorally Tailored inTERventions for You) is a Horizon Europe initiative (GAP 101080117) dedicated to advancing our understanding of the multifaceted etiology of obesity. The project aims to move beyond current knowledge of obesity‐related determinants by examining the system‐level interactions between biological (including genetics), lifestyle behaviors (physical activity, nutrition, sedentary behaviors), and contextual factors, including social, economic, psychological, and environmental factors. BETTER4U advances from traditional approaches by exploring determinants embedded within interconnected systems and biological frameworks. It seeks to identify and integrate polygenic risks, omics‐based markers, lifestyle and contextual factors using data from biobanks and previous landmark studies. This comprehensive approach enables the refinement of Artificial Intelligence (AI) algorithms, enhancing individualization in the design of tailored interventions. The project employs real‐time behavioral monitoring tools and remote technologies to track individual behaviors and metabolic responses, allowing for the development of personalized obesity prevention strategies. By doing so, BETTER4U aims to bridge the gap between research and practical application, enabling the design of interventions that are biologically and behaviorally informed. The anticipated outcomes of BETTER4U include advanced AI models that support the development of sophisticated, targeted interventions for individuals at risk of overweight or obesity. These models will provide actionable insights into how personalized lifestyle adjustments in lifestyle behaviors, such as diet and physical activity, can effectively mitigate weight gain trajectories across the lifespan. Ultimately, the project seeks to empower individuals and support precision medicine approaches in obesity prevention and treatment through active participant engagement.

## 1 Introduction

**FIGURE 1:**
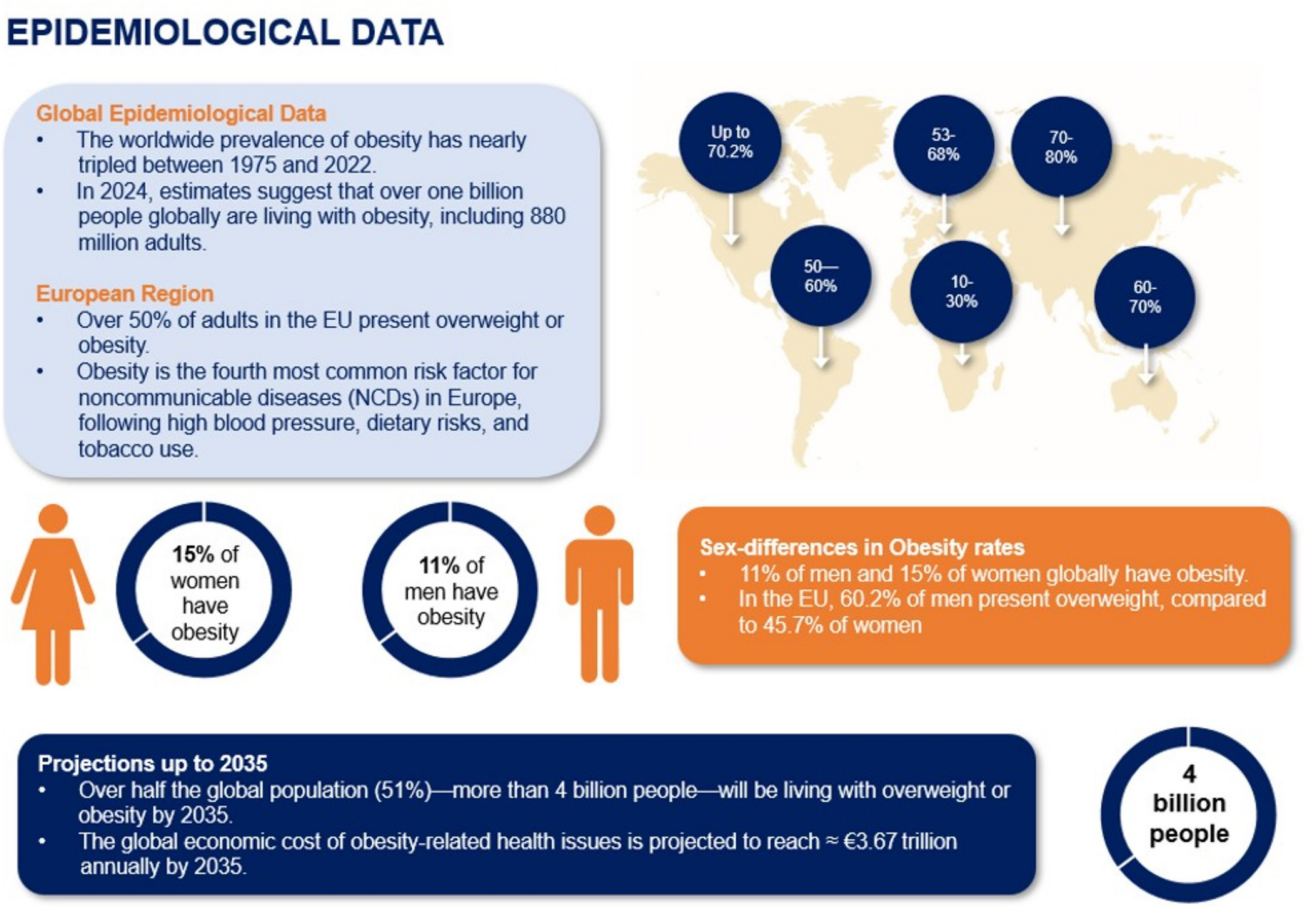
Epidemiological Data for Obesity. As of 2024, over one billion people—about 880 million adults—are living with obesity [1, 2]. Obesity affects up to 70.2% of the population in the United States, 50%–60% in South America, 53%–68% in Europe, 70%–80% in Asia, and 60%–70% in Australia, reflecting a widespread and growing global health challenge. Globally, 15% of women and 11% of men are affected, while in the EU, 60.2% of men and 45.7% of women present overweight, highlighting the importance of sex‐specific strategies [1, 2, 3, 4, 5]. By 2035, over half the world's population is expected to present overweight or obesity, with related healthcare costs projected at $4.32 ≈ €3.67 trillion annually [6].

Overweight and obesity are a critical and growing public health concern within the World Health Organization (WHO) European Region, contributing significantly to increased burden of NCDs, disability, and premature mortality [1]. Despite growing awareness, obesity rates have nearly tripled since 1975 [2]. In 2016, the WHO reported that more than 1.9 billion adults, i.e. approximately 40% of the global population, presented overweight, including over 650 million (13%) who were living with obesity [3]. By 2022, this number had risen to 2.5 billion adults, with 890 million living with obesity [4]. In the European Region, nearly 60% of adults are affected by overweight and obesity—with a higher prevalence among men (63%) than women (54%) [3, 4, 5] (Figure 1 showing the global prevalence of overweight and obesity). One in three school‐aged children is classified as having overweight or obesity, while the prevalence is 8% for children under five and one in four for adolescents [3, 4, 5]. Obesity prevalence is also disproportionately higher among individuals with lower educational attainment. Similar trends are observed among children based on parental education—especially in high‐income countries [7, 8].

Current projections indicate that overweight and obesity are associated with over 200 health‐related complications and are responsible for over 1.2 million deaths per year in the European region, ranking them as the fourth leading risk factor for other noncommunicable diseases (NCDs), after high blood pressure, dietary risks, and tobacco smoking [2, 6]. Costs linked to overweight or obesity amount to about US $425 billion among the Organization for Economic Co‐operation and Development (OECD), Group of Twenty (G‐20), Europe, and various partner countries (52 countries). According to the latest global data, the cost is even greater, estimated at around 2.2% of the global Gross Domestic Product (GDP) (~US $2 trillion/year) and potentially reaching US $4 trillion/year by 2035. Overweight and obesity impose a significant financial burden on healthcare facilities, expected to account for 60%–70% of diabetes‐related treatment costs, 20%–30% of cardiovascular treatment costs, and 5%–10% of cancer‐related healthcare expenditures [6, 9]. The OECD predicts that between 2020 and 2050, 8.4% of total healthcare spending will be necessary to manage overweight and its complications [6, 9].

Beyond the direct health implications, addressing obesity is fundamental to sustainable development, given its profound impact on equity and economic development [10]. While not explicitly named in the Sustainable Development Goals (SDG) framework, obesity intersects with goals such as SDG 3 (Good Health and Well‐Being), SDG 2 (Zero Hunger), and SDG 10 (Reduced Inequalities), as it contributes significantly to the global burden of noncommunicable diseases, coexists with food insecurity, and disproportionately affects lower‐income populations. Moreover, obesity undermines educational attainment (SDG 4), labor productivity (SDG 8), and sex equality (SDG 5) [10]. Integrated strategies to prevent and manage obesity—particularly those that address social determinants and structural drivers—are therefore critical to advancing both public health and sustainable development agendas.

Obesity is a multifactorial condition, influenced by both innate biological predispositions and modifiable behavioral and environmental factors. Genetic makeup, metabolic and lipidomic profiles, adipokine signaling, gut microbiome composition, dietary habits, physical activity levels, psychological well‐being, and environmental context all interact in complex ways [11, 12, 13, 14]. Over the last 20 years, Genome‐Wide Association Studies (GWAS) have identified multiple genetic pathways contributing to obesity, including those involved in hedonic eating, adipogenesis, angiogenesis, insulin resistance, and transcriptional regulation [15, 16, 17, 18]. Emerging evidence also highlights how genetic variation interacts with the gut microbiome and metabolic pathways, revealing distinct causal mechanisms underlying weight gain [19].

This growing body of research supports the use of integrated biological data—genetic, metabolomic, and microbiome—to enhance early risk assessment, forecast weight trajectories, and develop personalized dietary and behavioral interventions. Traditional “one‐size‐fits‐all” obesity strategies have shown limited effectiveness, reinforcing the urgent need for individualized approaches that consider variability in genetics, behavior, and environment [20]. Precision medicine and personalized nutrition strategies have shown encouraging results [21, 22]. For instance, a recent systematic review and meta‐analysis found that technology‐delivered personalized nutrition interventions led to significant improvements in dietary behavior and significant reductions in energy intake among individuals with overweight and obesity [23].

Clinical trials further demonstrate considerable inter‐individual variation in response to weight loss interventions, often influenced by complex, non‐linear interactions between biological, lifestyle, and contextual factors. Importantly, evidence suggests that while genetic predisposition increases susceptibility, its negative effects can be reduced by sustained healthy lifestyle behaviors, such as a healthy diet, regular physical activity, and reduced sedentary behaviors [24, 25, 26]. Therefore, personalized interventions, based on biological insight and tailored to each person's profile, are increasingly recognized as a potentially highly effective strategy for obesity prevention and treatment [24, 25, 26].

Technological advancements—particularly in digital health and AI—are revolutionizing obesity care. AI enables the analysis of large‐scale and diverse datasets, including electronic health records, wearable sensor outputs, meal images, and patient‐reported data, to detect patterns and predict individual responses to treatment [27]. This allows healthcare providers to design and adjust interventions in real time, improving efficacy and adherence. Additionally, AI‐powered remote monitoring tools enable continuous tracking of behavioral and physiological parameters, facilitating timely feedback and personalized support [28, 29]. These technologies empower patients to actively engage in their care and enable clinicians to make more informed decisions, ultimately enhancing health outcomes.

In conclusion, integrating personalized interventions with AI and remote monitoring technologies is essential for effectively addressing the complex, multifactorial nature of obesity. By combining scientific insights from genetics and behavior with cutting‐edge technological tools, health systems can develop more precise, responsive, and efficient strategies for preventing and treating obesity, further contributing to broader public health goals of health equity and sustainable development. Within this framework, the BETTER4U project aims to advance tailored interventions for obesity by integrating omics data, lifestyle behaviors' monitoring, and AI‐driven personalization.

## 2 The BETTER4U Project

### 2.1 BETTER4U Project Vision and Objectives

The uniqueness of BETTER4U lies in several aspects that distinguish it from other large‐scale projects. It is pioneering in aspiring to integrate such extensive datasets or previous cohorts, including data from approximately 1 million individuals, including genetics, lifestyle, biochemical, clinical, omics, and microbiome data. BETTER4U is also the first to combine genetic risk information with digital technologies, real‐time monitoring, and lifestyle interventions, incorporating polygenic risk scores (PRS) directly into an AI‐based causal model to generate personalized recommendations. Moreover, it practically builds upon and complements the findings of previous initiatives, expanding and integrating their insights in a pragmatic and holistic approach.

**FIGURE 2:**
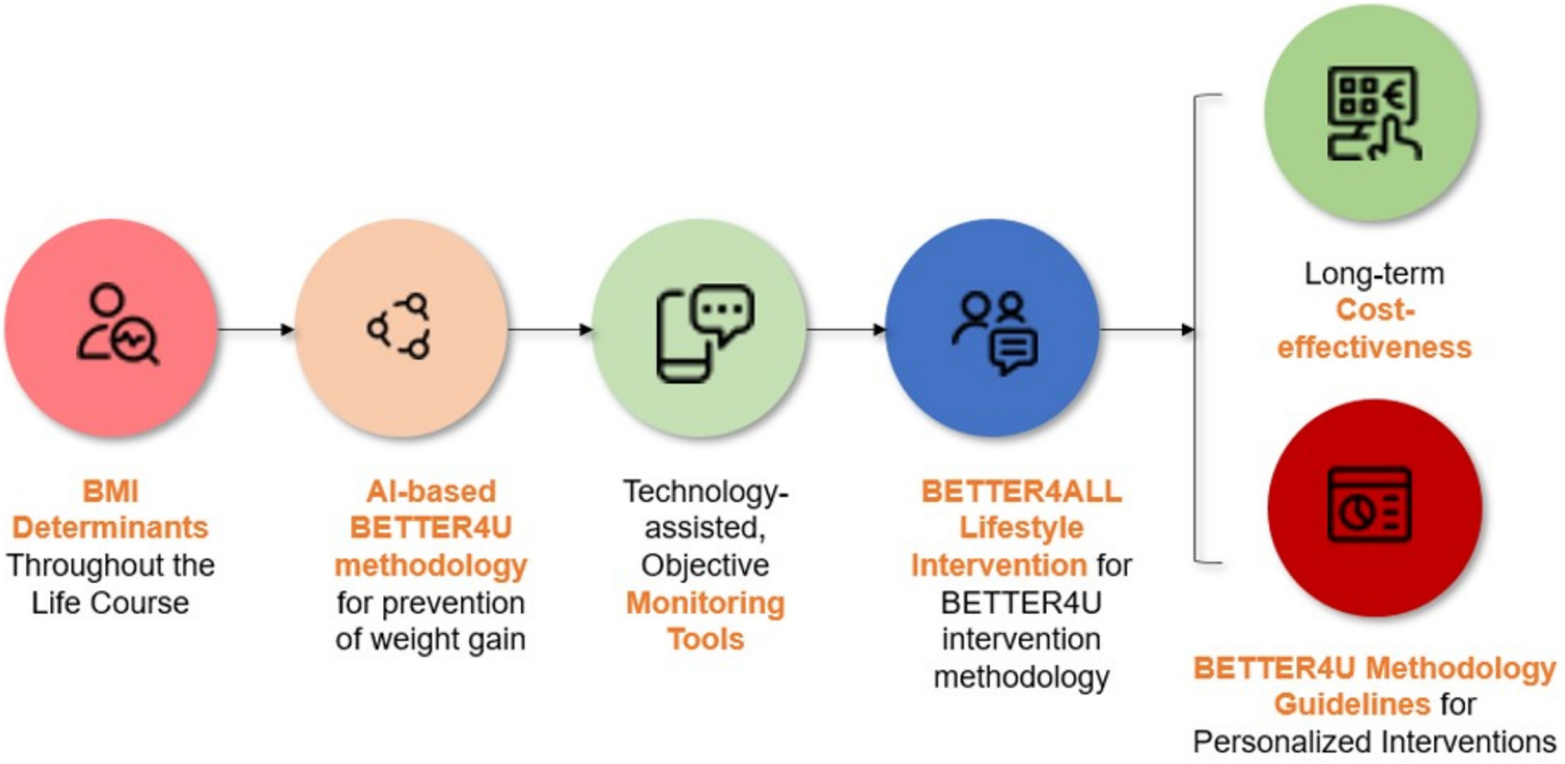
BETTER4U Workflow (created with BioRender.com).

The BETTER4U project includes 28 partners focusing on obesity prevention and management using a systemic, individualized approach that considers biological elements together with behavioral aspects and environmental factors. As shown in Figure 2, BETTER4U workflow is summarized in first using data from an extensive pool of existing cohorts contributed to the project and forming the BETTER4U dataset to analyze the complex interactions between genetics, lifestyle, and environmental influences on body mass index (BMI) throughout different stages of life (*Objective 1*). BMI was chosen as the primary marker of obesity due to its widespread use and universality in identifying obesity, as well as its availability across most cohorts and datasets, including those from vulnerable populations where detailed body composition data may not be accessible. However, due to the limitations of BMI in capturing body fat distribution, body composition measurements will also be collected, analyzed, and included to complement BMI and provide a more comprehensive assessment of obesity. Leveraging this knowledge, BETTER4U will create an AI‐driven algorithm designed to pinpoint critical obesity factors and customize individual prevention plans (*Objective 2*). The project proposes using state‐of‐the‐art monitoring devices that track behaviors and contextual elements continuously resulting in the provision of real‐time feedback for dynamically adaptive intervention strategies (*Objective 3*). The novelty of this approach will first be assessed in a pilot study across seven European sites, followed by the BETTER4ALL lifestyle intervention via a Randomized Controlled Trial (RCT) where participants will receive tailored recommendations through an AI‐based causal model (*Objective 4*). Besides clinical evaluations, BETTER4U aims to analyze the long‐term costs and budgetary impacts of interventions devised by BETTER4U methodology in order to facilitate wide‐scale implementation. This is captured in BETTER4U's fifth objective: the large‐scale implementation of BETTER4U's findings while supporting sustainable adoption through cost‐effective interventions—making the program economically attractive for various stakeholders (*Objective 5*). Lastly, the project's collaborative strategy includes translating its findings into actionable policy briefs with key public and private stakeholders to support rapid dissemination across seven European countries which would help overcome existing silos limiting cross‐border cooperation in Europe (*Objective 6*).

## 3 Methods and Design

**FIGURE 3:**
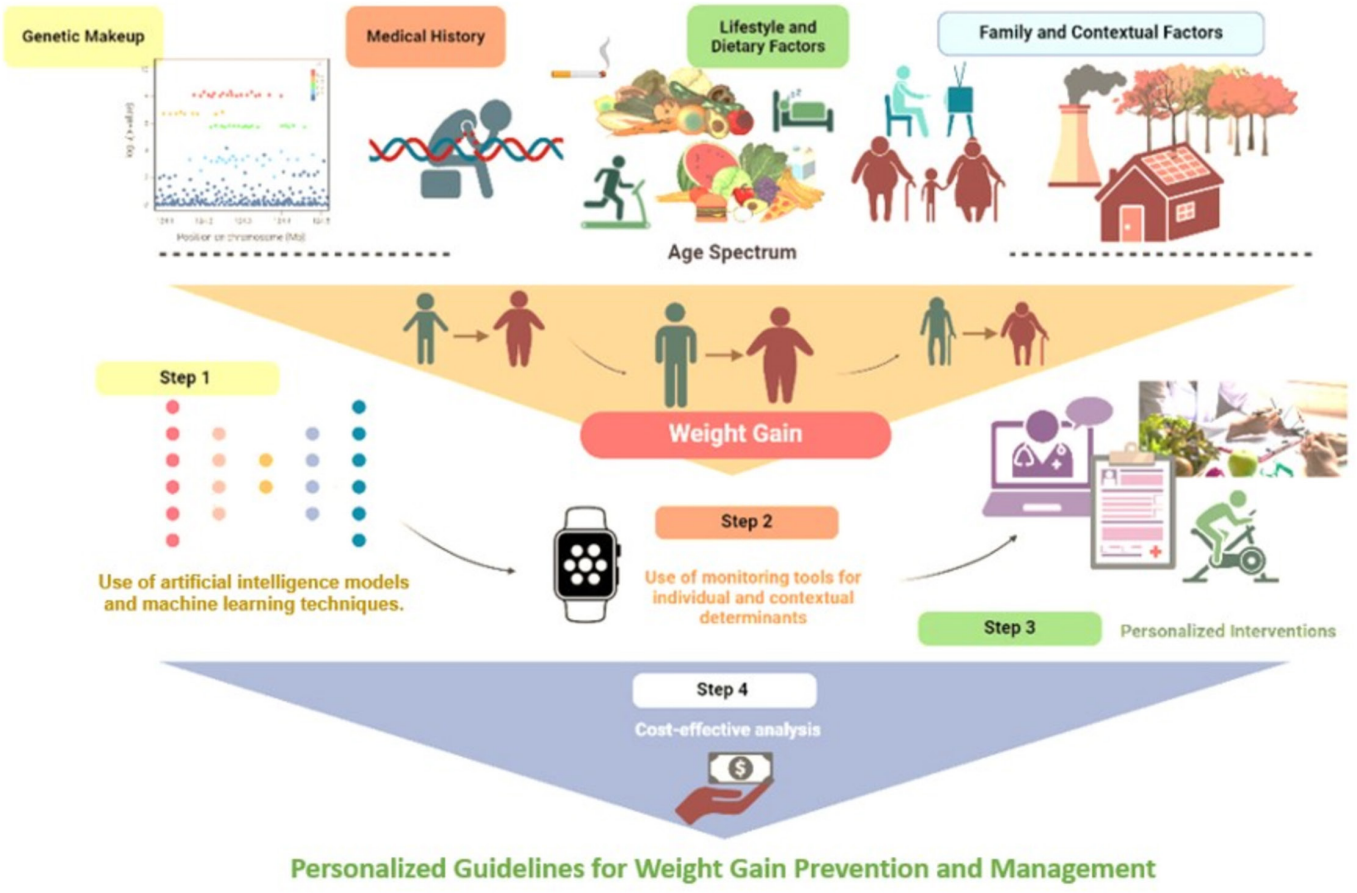
Structure of BETTER4U Implementation Phases (created with BioRender.com).

**FIGURE 4:**
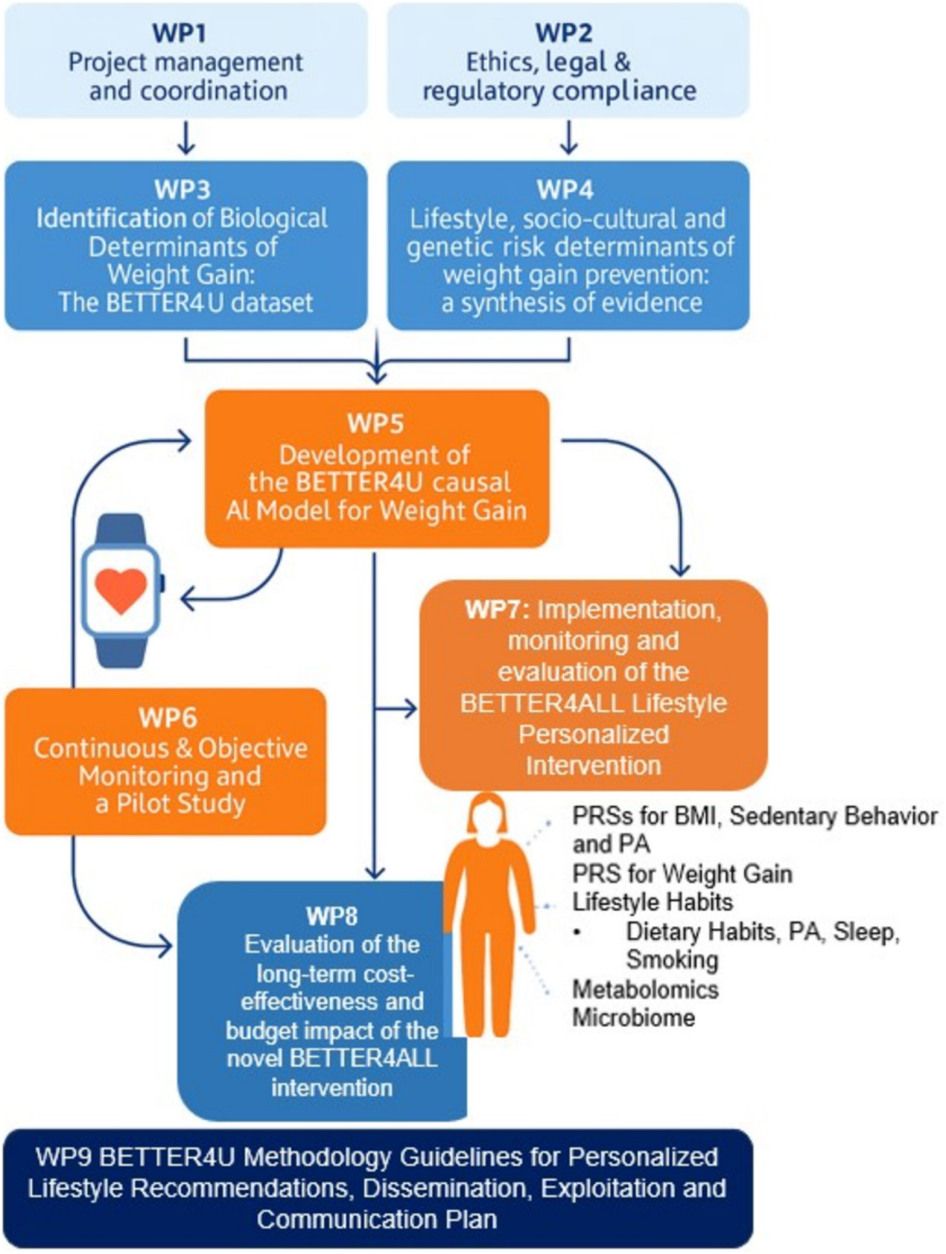
Work Package (WP) Structure of the BETTER4U project.

BETTER4U is structured across four implementation phases to enable the logical cohesion of determinant identification, their subsequent use for predictive modeling of AI‐derived, tailored interventions, and the scalability of the overall proposed BETTER4U methodology for personalized interventions (Figure 3). ***Phase 1*** lays the scientific foundation of BETTER4U by uncovering the multifaceted causes of weight gain through a combination of large‐scale data analysis and state‐of‐the‐art bioinformatics. It begins with the launch of work package (**WP)3** and **WP4** (Figure 4), which focus on identifying the biological and lifestyle determinants of weight gain. This phase includes a thorough meta‐review of existing literature alongside large‐scale meta‐analyses using the BETTER4U dataset, an integrated resource of over 1 million individual‐level records across the EU and internationally, including data from large‐scale projects and cohorts (e.g., Active Dyads: Partner—Partner, Active Dyads: Parent–Child, Active Action, BIONIC cohorts, Born in Bradford—BiB, Big data against childhood obesity—BigO, the Childhood Obesity Surveillance Initiative—COSI, the Copenhagen Prospective Studies on Asthma in Childhood—COPSAC, Estonian Biobank (EstBB), Feel4Diabetes, Finnish Twin Cohorts—FTC, GINIplus&LISA birth cohorts, Health Professionals Follow‐Up Study—HPFS, Harokopio University of Athens BioResource, Healthy Growth Study, Netherlands Twin Register—NTR, Nurses' Health Study—NHS, Nutrition Health Alliance—NutriHeal, Seguimient Univesity of Navarra—SUN project, biobank.cy, UK Biobank—UKBB, Young Finns Study—YFS, 10K project). The BETTER4U dataset will include genetic, lifestyle, behavioral and omics data available for the participants of the respective cohorts and used within WP3 and WP4 as per the needs of the corresponding analyses.

The main goal is to identify key genetic, metabolomic, lipidomic, adipokine, microbiome, and lifestyle factors, such as diet, physical activity, sedentary behavior, smoking, sleep, and socioeconomic status, that contribute to weight gain throughout life stages (childhood, adolescence, and adulthood), and to examine how these factors change with age.

Data analysis will be conducted using a federated approach that enables local data processing and harmonization across cohorts. Central objectives of Phase 1 include

- the conduct of a GWAS analysis for baseline weight/BMI and weight/BMI change using the BETTER4U dataset;
- the quantification of the genetic predisposition for BMI by replicating Polygenic Risk Scores (PRSs) retrieved from the previous literature [30];
- the development of a PRS for weight gain trajectories using data from the BETTER4U dataset;
- the identification of phenotypic, metabolomics, and socioeconomic determinants of weight gain;
- the determination of behavioral techniques maximizing intervention impact for weight management; and vi) the conduct of gene–lifestyle factors interactions studies to inform the creation of a Lifestyle Risk Score (LRS), which, when combined with genetic data, will further serve as input for AI‐driven models that deliver precision‐targeted recommendations for obesity prevention and management.

Analyzing patterns and drivers of weight gain provides critical insights into the early stages of excess weight accumulation, enabling timely intervention before obesity develops. These findings will inform the design of more effective, targeted prevention and management strategies by identifying key risk periods, populations, and modifiable factors.

In parallel, phenome‐wide association studies for a series of obesity‐related diseases and Mendelian randomization analyses will investigate the relationships between genetic risk for weight gain and various health outcomes. Machine learning (ML) techniques, including phenotype–genotype many‐to‐many relational analysis (PGMRA), will be applied to detect the complex genetic‐phenotypic associations, deepening our understanding of obesity's biological mechanisms. Furthermore, the attempt to disentangle the genetic etiology of weight gain will be further strengthened through the analysis of twin data included in the CODATwins [31] providing additional insights into the heritability and genetic underpinnings of weight gain. Additionally, systematic reviews of obesity management techniques in vulnerable populations will be conducted, including individuals of lower socioeconomic status, in order to inform the design of the subsequent phases.

In ***Phase 2***, the focus is on the development of the BETTER4U AI‐based, causal model for weight gain. This personalized intervention model incorporates causal knowledge in the form of Directed Acyclic Graphs (DAGs) and integrates advanced ML algorithms to discover functional and causal dependencies between genetics, biology, lifestyle choices, and environmental components. The process starts with the synchronization of local datasets along with preprocessing tasks, which include encoding, outlier removal, and imputation. The DAGs that will be utilized in guiding model construction have been informed from the analyses conducted in WP3 and WP4.. Models developed across sites will be integrated using federated learning on NVIDIA's Flare platform [32], allowing collaborative training without sharing sensitive data (***WP5***).

The integrated model shall then undergo multi‐level validation involving comprehensive cross‐validation within datasets as well as leave‐one‐dataset‐out‐testing and validation against interventional data. To continuously improve forecasting, the model will use future information from passive monitoring tools (***WP6***) and adherence data from the pilot study (e.g., estimating weight outcomes under different intervention scenarios). In its final form, the AI‐based, causal model will be integrated into a decision‐support system for dietitians allowing them to simulate and optimize patient‐specific recommendations. The system will undergo continual refinement through active learning as more individual‐level data becomes accessible. Data challenges, such as data inconsistency, ambiguous causal relationships, reasoning beyond the sample population, and outside relevance, will be addressed using methods allow learning from data stored in different locations without moving it, while also using patterns learned from similar datasets and the available twin data to better understand cause‐and‐effect relationships.

In ***Phase 3***, the focus is on developing comprehensive lifestyle and health metrics for objective monitoring using mobile and wearable sensor technologies (***WP6***) that are seamless and unobtrusive. These metrics will facilitate personalized behavior evaluations, monitor intervention compliance and effectiveness, and provide feedback to enhance participant engagement and self‐monitoring. To finalize the BETTER4ALL lifestyle intervention design, a pilot study with 490 participants (both end‐users and implementers) from seven European countries will be conducted. This study aims to further refine the causal AI model developed in Phase 2 by integrating it with real‐time‐streaming data. Enrollment will be done in cohorts, which will enable iterative refinements of not just the intervention protocol, but also the AI‐powered algorithms based on compliance behavior data. A dedicated intervention platform for implementers (i.e., the BETTER4U intervention platform), along with a mobile app, will be used for the continuous assessment throughout the intervention, facilitating real‐time data collection, tracking and communication. The pilot study shall identify and continuously assess Core Behavioral as well as Living Environment Indicators (BCBIs and LEIs, respectively) for subsequent use in the AI‐tailored recommendations in ***WP7***.

**FIGURE 5:**
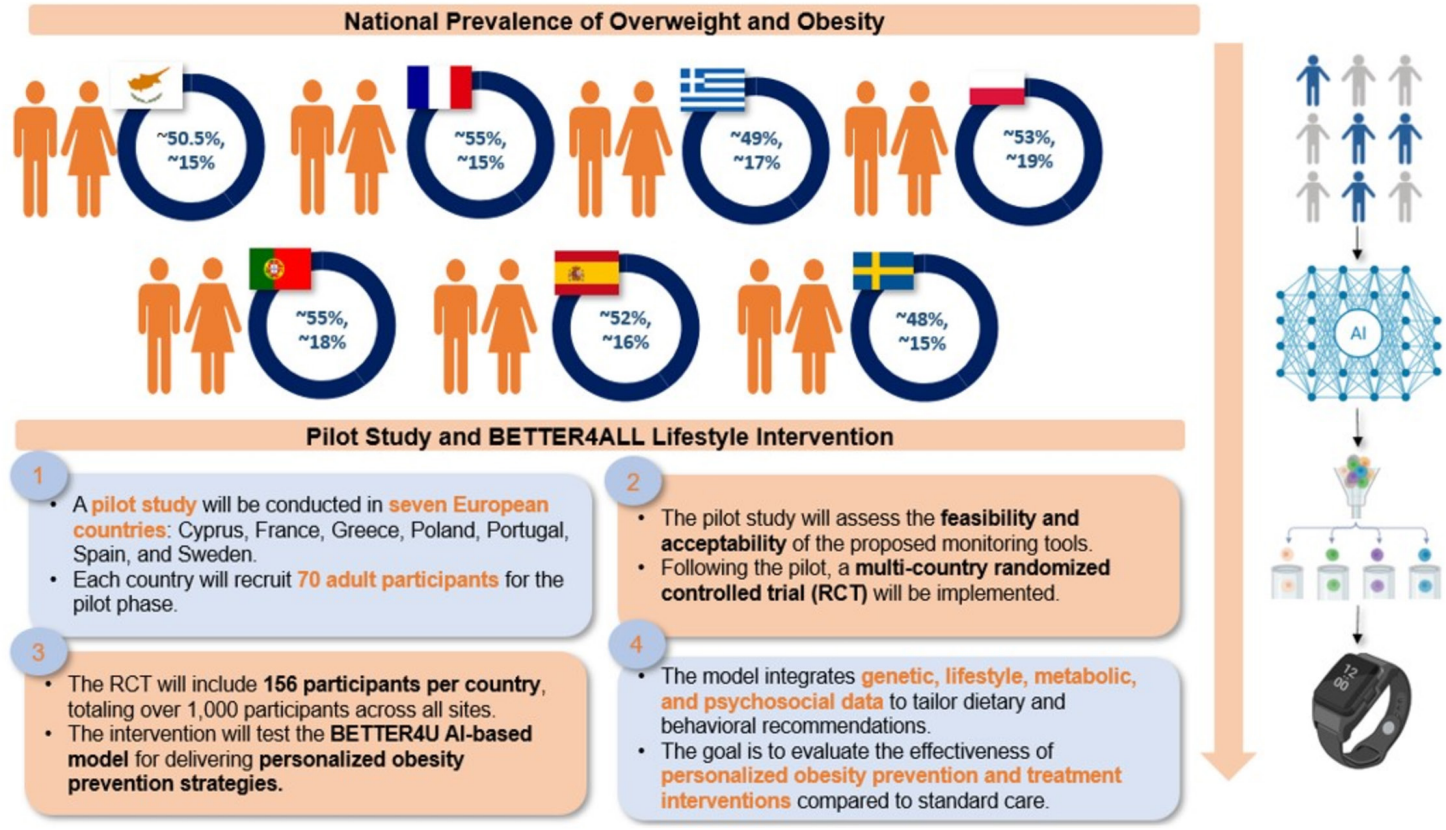
Pilot Study and BETTER4ALL Lifestyle Intervention. This figure outlines the structure of the pilot study and subsequent multi‐country RCT evaluating AI‐driven, personalized obesity prevention strategies. The pilot phase will run in seven European countries (Cyprus, France, Greece, Poland, Portugal, Spain, Sweden), with 70 adult participants per site, assessing the feasibility of technology‐enhanced monitoring tools. Pie charts above each country icon show national overweight and obesity rates, reflecting diverse health profiles. The RCT will then enroll 156 participants per country (over 1000 total) to test the BETTER4U AI‐based, causal model, which combines genetic, lifestyle, metabolic, and psychosocial data to deliver tailored interventions via digital tools like wearables. The trial aims to assess both the effectiveness and scalability of this approach versus standard obesity care.

Using this foundation, the latter will deliver personalized lifestyle interventions using the causal model, which will allow healthcare professionals to capture detailed individual demographic, genetic, biochemical, and lifestyle information for tailored dietary and behavioral recommendations powered by AI. These recommendations will provide continuous monitoring adjustments to ensure responsive and personalized care. This strategy will be systematically assessed through a six‐month RCT within the same seven countries with 1015 adults with overweight or obesity (Figure 5). The trial will test the provision of personalized recommendations against standard care using a hybrid digital approach through the mobile application alongside in‐person support. Primary outcomes include change in BMI, while secondary outcomes include body composition, cardiometabolic risk factors, metabolomic and microbiome profiles, quality of life, and downstream behavioral changes among household members. Additional assessments will focus on appetite control and weight maintenance over 12 months. Regional community partners will be engaged as active recruiters and adopters of BETTER4U principles. The RCT will be conducted across multiple European countries to ensure applicability in heterogeneous cultural contexts. Cultural differences, including dietary habits and other lifestyle characteristics, are explicitly incorporated into the AI‐based causal model, allowing the personalized RCT recommendations to be tailored to each participant's specific context. To prevent digital exclusion, smartwatches will be provided to participants, along with analytical guidelines to ensure digital literacy and reduce any risk of digital exclusion, both during the pilot study and the RCT.

Ultimately, **Phase 4** focuses on evaluating the cost‐effectiveness and budget impact of the BETTER4ALL lifestyle intervention approach in all implementation countries (***WP8***). In order to evaluate the additional costs per quality‐adjusted life years (QALYs) gained from the perspective of the healthcare payers, the results of the one‐year study will be extrapolated over a lifetime horizon using advanced decision analytic approaches. Sensitivity analyses will account for uncertainty in the results and identify cost‐effectiveness drivers. Additionally, a budget impact analysis will assess the financial consequences of implementing the intervention. Both health economic evaluations will inform healthcare policy‐ and decision makers regarding resource allocation decisions and support considerations for large‐scale implementation.

To boot, phase 4 includes the creation and distribution of methodological guidelines and communication strategies in addition to the economic evaluation (WP9). Through focused workshops, awareness campaigns, and distribution through European Commission channels, these initiatives seek to spread knowledge of BETTER4U's tailored lifestyle advice among stakeholders, including healthcare professionals, researchers, and citizens, through presentations, workshops, and social dissemination to promote science literacy, collect feedback, and ensure transparent management of research outputs. For this purpose, an open‐access BETTER4U hub on the Zenodo platform will be created to share tools and support stakeholder engagement, co‐creation, and collaboration with related EU and national projects. This platform will help ensure the project's findings are accessible and implemented effectively. BETTER4U will implement and abide by all relevant Open Science practices by digital sharing of its data, models, and outcomes that can be used for validation and reproducibility. The project will share curated and anonymized datasets through public repositories like Zenodo, adhering to ethical, privacy, and legal constraints. The project will allow public access to its AI‐based causal models and measurement algorithms, thereby facilitating open evaluation and setting a baseline for future research. In addition,

Ultimately, phase 4 focuses on evaluating the cost‐effectiveness and budget impact of the BETTER4ALL lifestyle intervention approach (WP8) in all implementation countries. In order to evaluate the additional costs per quality adjusted life year (QALY) gained from a healthcare payer perspective, the results of the one‐year study will be extrapolated over a lifetime horizon using an advanced decision‐analytic model. Sensitivity analyses will account for uncertainty in the results and identify cost‐effectiveness drivers. Additionally, a budget impact analysis will assess the financial consequences of implementing the intervention. Both health economic evaluations will inform healthcare policy and decision makers regarding resource allocation and affordability efficiency which will support considerations for large‐scale implementation.

## 4 Discussion

Obesity remains a major global health challenge, contributing substantially to morbidity, mortality, and healthcare costs worldwide [33]. Despite advances in pharmacological treatments and public health initiatives, sustainable long‐term weight management remains elusive for many individuals. This gap underscores the pressing need for innovative, integrative approaches that combine pharmacological interventions with tailored lifestyle modifications, guided by emerging technologies such as AI. The BETTER4U project represents a pioneering effort to harness AI‐driven personalized recommendations to modernize obesity care by integrating causal modeling, real‐time monitoring, and adaptive intervention strategies.

Traditional obesity interventions frequently apply a one‐size‐fits‐all approach that neglects the complex interplay of genetic, metabolic, behavioral, and environmental factors influencing individual susceptibility to weight gain and response to treatment [34]. Personalized medicine, leveraging data‐driven AI models, promises to revolutionize this landscape by enabling precision targeting of interventions based on an individual's unique biological and lifestyle profile [35].

BETTER4U aims to provide methodological guidelines to support the implementation of personalized recommendations for individuals living with overweight or obesity across diverse European health systems. By including cohorts from multiple European countries, the AI‐driven recommendations account for geographical and phenotypic variations, allowing the guidelines to be tailored to each participant regardless of location. Given the recent pharmacological advances and while the project does not directly include such interventions, its methodology is designed as such to be able to take into account participants' pharmacological characteristics and use them to tailor recommendations that can integrate and support ongoing medical treatments, ensuring an element of reciprocal complementarity. This holistic approach, emphasizing the role of lifestyle parameters and genetic risk, ensures long‐term sustainability and reduces relapse risk, even when pharmacological strategies are employed.

BETTER4U's AI‐based, causal model stands out by combining established causal frameworks with ML techniques to quantify functional and causal relationships among variables influencing weight change. This methodological sophistication allows for counterfactual predictions—estimating outcomes of hypothetical interventions—which is critical for tailoring lifestyle recommendations effectively [36]. By incorporating PRSs, behavioral indicators, and continuous passive monitoring data, BETTER4U advances beyond static risk prediction to dynamic, context‐sensitive intervention adaptation.

### 4.1 Integrating AI Recommendations With Pharmacological and Lifestyle Approaches

Recent years have seen rapid development in obesity pharmacotherapy, with agents such as GLP‐1 receptor agonists demonstrating significant weight reduction and cardiometabolic benefits [37]. However, pharmacological treatment alone is often insufficient without complementary lifestyle changes—including diet, physical activity, and behavioral support, which are essential to sustain weight loss and prevent relapse [38]. The BETTER4U approach aligns with this interdisciplinary paradigm by providing healthcare professionals with AI‐driven decision support tools that integrate biological data, treatment adherence, and lifestyle behaviors. This facilitates the development of optimized intervention plans tailored to the individual's risk profile and response patterns, potentially enhancing pharmacotherapy efficacy and patient engagement. Furthermore, the continuous monitoring enabled by wearable sensors and mobile applications allows real‐time feedback loops that can detect early signs of non‐compliance or plateauing, prompting timely adjustments to the treatment plan.

### 4.2 Addressing Barriers and Promoting Scalability Through Implementation Science

Translating AI‐driven personalized interventions into routine clinical practice faces multiple challenges, including data heterogeneity, privacy concerns, clinician training, and patient acceptability [39]. BETTER4U proactively incorporates implementation science frameworks such as the Consolidated Framework for Implementation Research (CFIR) [40] and Context and Implementation of Complex Interventions (CICI) [41] to evaluate barriers and facilitators across diverse European contexts, increasing the likelihood of successful deployment and scalability. Moreover, the use of federated learning within BETTER4U's model development addresses data privacy by enabling model training across distributed datasets without centralized data sharing [42]. This innovative approach promotes inclusivity and generalizability, crucial for capturing population heterogeneity in obesity phenotypes and socio‐cultural influences on health behaviors.

### 4.3 Potential Impact on Health Outcomes and Health Systems

The BETTER4U project is highly relevant for the community as it aims to prevent and manage obesity through biologically tailored interventions that account for individual differences in genetics, metabolism, and lifestyle. By integrating scientific research with practical, community‐based approaches, BETTER4U empowers individuals, especially those in vulnerable populations, to make informed health decisions. The project also fosters collaboration between researchers, healthcare providers, and policymakers to develop effective, personalized strategies that support long‐term health and reduce the burden of obesity‐related diseases at the population level.

The integration of AI‐driven personalized recommendations within obesity care pathways holds promise for improving long‐term outcomes, including sustained weight loss, metabolic risk reduction, and quality of life [16]. By optimizing intervention intensity and content at the individual level, BETTER4U could reduce the burden of ineffective treatments and avoidable complications, thus enhancing health system efficiency and cost‐effectiveness. Furthermore, the project's comprehensive evaluation of cost‐effectiveness and budget impact is critical to support policy decisions regarding reimbursement and wider adoption. As healthcare systems worldwide grapple with resource constraints, evidence of value‐for‐money will be paramount for scaling AI‐powered personalized obesity interventions [43].

### 4.4 Future Directions and Research Needs

Despite promising advancements, several knowledge gaps remain. In order to begin to show predictive accuracy and clinical utility longitudinally, AI‐based models would need to be validated in diverse populations and other real‐world contexts. Ethical issues associated with data use, privacy, algorithm transparency, and equitable access to personalized interventions are ongoing concerns to continue to monitor [41]. Within this evolving landscape, the BETTER4U project offers insights that extend beyond the original framework or aim of the study. It illustrates how a comprehensive design that integrates biological (including genetics), lifestyle behaviors (physical activity, nutrition, sedentary behaviors), and contextual factors can be incorporated into population‐level health engagements to support more targeted and impactful approaches to obesity prevention. By prioritizing vulnerable populations, BETTER4U underscores the importance of equity in innovation, ensuring that personalized interventions are both accessible and contextually relevant across diverse socioeconomic settings. Moreover, its tools—the AI‐based models, the PRS for weight gain, the LRS, and the tailored intervention and monitoring tools—shall lay the groundwork for scalable, adaptable frameworks for future health programs. Ultimately, BETTER4U exemplifies how precision health can be leveraged to achieve inclusive and sustainable public health impact. Future research should build on this foundation by deepening the integration of genetics, −omics, and lifestyle data with environmental and psychosocial factors, further refining predictive models and enhancing the personalization of care.

## Funding

The BETTER4U project has received funding from the European Union's Horizon Europe Research and Innovation program under Grant Agreement No. 101080117. Additionally, BETTER4U is funded by UK Research and Innovation (UKRI) under the UK government's Horizon Europe funding guarantee (grant number 10093560 for QMUL and 10106435 for BiB) and by the Swiss State Secretariat for Education, Research and Innovation (SERI).

## “On behalf of the BETTER4U Consortium” Members List:

George Dedoussis, Department of Nutrition and Dietetics, School of Health Science and Education, Harokopio University of Athens, 17676 Athens, Greece, Genome Analysis, 17676 Athens, Greece

Yannis Manios, Department of Nutrition and Dietetics, School of Health Science and Education, Harokopio University of Athens, 17676 Athens, Greece. European Centre for Obesity, Harokopio University of Athens, 17676 Athens, Greece, Institute of Agri‐Food and Life Sciences, Hellenic Mediterranean University Research Centre, 71410 Heraklion, Greece

Christos Diou, Department of Informatics and Telematics, School of Digital Technology, Harokopio University of Athens, 17778 Athens, Greece

Panagiotis Moulos, Department of Nutrition and Dietetics, School of Health Science and Education, Harokopio University of Athens, 17676 Athens, Greece

Ioanna Panagiota Kalafati, Department of Nutrition and Dietetics, School of Health Science and Education, Harokopio University of Athens, 17676 Athens, Greece

Maria Kafyra, Department of Nutrition and Dietetics, School of Health Science and Education, Harokopio University of Athens, 17676 Athens, Greece

Panagiotis Symianakis, Department of Nutrition and Dietetics, School of Health Science and Education, Harokopio University of Athens, 17676 Athens, Greece.

Eva Karaglani, Department of Nutrition and Dietetics, School of Health Science and Education, Harokopio University of Athens, 17676 Athens, Greece.

Despina Brekou, Department of Nutrition and Dietetics, School of Health Science and Education, Harokopio University of Athens, 17676 Athens, Greece.

Vasiliki Vavouraki,

Department of Nutrition and Dietetics, School of Health Science and Education, Harokopio University of Athens, 17676 Athens, Greece, Genome Analysis, 17676 Athens, Greece

Christina Patmiou, Department of Nutrition and Dietetics, School of Health Science and Education, Harokopio University of Athens, 17676 Athens, Greece

Paris Kantaras, Department of Nutrition and Dietetics, School of Health Science and Education, Harokopio University of Athens, 17676 Athens, Greece

Panagiotis Alimisis, Department of Informatics and Telematics, School of Digital Technology, Harokopio University of Athens, 17778, Greece.

Anastasios Papamanolis, Department of Informatics and Telematics, School of Digital Technology, Harokopio University of Athens, 17778, Greece.

Ana Rito, Centro de Estudos e Investigacão em Dinâmicas Sociais e Saúde, 1649–016 Lisbon, Portugal.

Marta Gaspar, Centro de Estudos e Investigacão em Dinâmicas Sociais e Saúde, 1649–016 Lisbon, Portugal.

Fátima Martins, Centro de Estudos e Investigacão em Dinâmicas Sociais e Saúde, 1649–016 Lisbon, Portugal.

Julie‐Anne Nazare, Centre de Recherche en Nutrition Humaine Rhône‐Alpes, Univ‐Lyon, CarMeN Laboratory, Inserm U1060, INRAE UMR1397, Université Claude Bernard Lyon 1, Pierre Bénite, France.

Louise Seconda, Centre de Recherche en Nutrition Humaine Rhône‐Alpes, Univ‐Lyon, CarMeN Laboratory, Inserm U1060, INRAE UMR1397, Université Claude Bernard Lyon 1, Pierre Bénite, France.

Anestis Dougkas, Lyfe Institute, Ecully, France and Centre de Recherche en Nutrition Humaine Rhône‐Alpes, Univ‐Lyon, CarMeN Laboratory, Inserm U1060, INRAE UMR1397, Université Claude Bernard Lyon 1, Pierre Bénite, France.

Klaus Bønnelykke, Copenhagen Prospective Studies on Asthma in Childhood, COPSAC, Region Hovedstaden 2870 Gentofte, Denmark.

Rebecca Kofod Vinding, Copenhagen Prospective Studies on Asthma in Childhood, COPSAC, Region Hovedstaden 2870 Gentofte, Denmark.

David Horner, Copenhagen Prospective Studies on Asthma in Childhood, COPSAC, Region Hovedstaden, 2870 Gentofte, Denmark.

Stephan Kampshoff, Science Communication Department, European Food Information Council (EUFIC), 1040 Brussels, Belgium.

Darya Silchenko, Science Communication Department, European Food Information Council (EUFIC), 1040 Brussels, Belgium.

Maria Hassapidou, Department of Nutritional Sciences & Dietetics, School of Health Sciences, International Hellenic University, 57400 Thessaloniki, Greece.

Ioannis Pagkalos, Department of Nutritional Sciences & Dietetics, School of Health Sciences, International Hellenic University, 57400 Thessaloniki, Greece.

Elena Patra, Department of Nutritional Sciences & Dietetics, School of Health Sciences, International Hellenic University, 57400 Thessaloniki, Greece.

Ioannis Ioakeimidis, Department of Medicine Huddinge (MedH), Karolinska Institutet, 171 77 Stockholm, Sweden.

Anna Ek, Department of Clinical Science, Intervention and Technology (CLINTEC), Karolinska Institutet, 171 77, Stockholm, Sweden.

Alkyoni Glympi, Department of Medicine Huddinge (MedH), Karolinska Institutet, 171 77 Stockholm, Sweden.

Eva Schernhammer, Department of Epidemiology, Center for Public Health, Medical University of Vienna, 1090 Vienna, Austria.

Magdalena Zebrowska, Department of Epidemiology, Center for Public Health, Medical University of Vienna, 1090 Vienna, Austria.

Constantinos Deltas, biobank.cy, Center of Excellence in Biobanking and Biomedical Research, University of Cyprus, 2109 Nicosia, Cyprus.

Panagiota Veloudi, biobank.cy, Center of Excellence in Biobanking and Biomedical Research, University of Cyprus, 2109 Nicosia, Cyprus.

Alexis Kyriacou, biobank.cy, Center of Excellence in Biobanking and Biomedical Research, University of Cyprus, 2109 Nicosia, Cyprus.

Apostolos Malatras, biobank.cy, Center of Excellence in Biobanking and Biomedical Research, University of Cyprus, 2109 Nicosia, Cyprus.

Jaakko Kaprio, Institute for Molecular Medicine Finland FIMM, HiLIFE, University of Helsinki, FI‐00014 Helsinki, Finland.

Teemu Palviainen, Institute for Molecular Medicine Finland FIMM, HiLIFE, University of Helsinki, FI‐00014 Helsinki, Finland.

Karri Silventoinen, Population Research Unit, Faculty of Social Sciences, University of Helsinki, FIN‐00014, Helsinki, Finland.

Alvaro Obeso, Population Research Unit, Faculty of Social Sciences, University of Helsinki, FIN‐00014, Helsinki, Finland.

Maira Bes‐Rastrollo, Department of Preventive Medicine and Public Health, University of Navarra; CIBERobn; Navarra Institute for Health Research (IdiSNA), 31008 Pamplona, Spain.

Carmen Sayon‐Orea, Department of Preventive Medicine and Public Health, University of Navarra; CIBERobn; Navarra Institute for Health Research (IdiSNA), 31008 Pamplona, Spain.

Miguel A. Martinez‐Gonzalez, D Department of Preventive Medicine and Public Health, University of Navarra; CIBERobn; Navarra Institute for Health Research (IdiSNA), 31008 Pamplona, Spain.

Cristina Razquin, Department of Preventive Medicine and Public Health, University of Navarra; CIBERobn; Navarra Institute for Health Research (IdiSNA), 31008 Pamplona, Spain.

Vanessa Bullon‐Vela, Department of Preventive Medicine and Public Health, University of Navarra, 31008 Pamplona, Spain.

Delfien Gryspeerdt, Department of Public Health and Primary Care, Interuniversity Centre for Health Economics Research (I‐CHER), Ghent University, 9000 Ghent, Belgium.

Nick Verhaeghe, Department of Public Health and Primary Care, Interuniversity Centre for Health Economics Research (I‐CHER), Ghent University, 9000 Ghent, Belgium.

Ruben Willems, Department of Public Health and Primary Care, Interuniversity Centre for Health Economics Research (I‐CHER), Ghent University, 9000 Ghent, Belgium.

Lieven Annemans, Department of Public Health and Primary Care, Interuniversity Centre for Health Economics Research (I‐CHER), Ghent University, 9000 Ghent, Belgium.

Aleksandra Luszczynska, Institute of Psychology, CARE‐BEH Center for Applied Research on Health Behavior and Health, SWPS University, 03815 Warsaw, Poland.

Paulina Krzywicka, Institute of Psychology, CARE‐BEH Center for Applied Research on Health Behavior and Health, SWPS University, 03815 Warsaw, Poland.

Zofia Szczuka, Institute of Psychology, CARE‐BEH Center for Applied Research on Health Behavior and Health, SWPS University, 03815 Warsaw, Poland.

Hanna Zaleskiewicz, Institute of Psychology, CARE‐BEH Center for Applied Research on Health Behavior and Health, SWPS University, 03815 Warsaw, Poland.

Anna Kornafel, Institute of Psychology, CARE‐BEH Center for Applied Research on Health Behavior and Health, SWPS University, 03815 Warsaw, Poland.

Anders Eriksson, Institute of Genomics, University of Tartu, 51010 Tartu, Estonia.

Elisabeth Thiering, Institute of Epidemiology, Helmholtz Zentrum München, German Research Center for Environmental Health, 85764, Neuherberg, Germany.

Marie Standl, Institute of Epidemiology, Helmholtz Zentrum München, German Research Center for Environmental Health, 85764, Neuherberg, Germany.

Tamara Schikowski, IUF—Leibniz Research Institute for Environmental Medicine.

Gunda Herberth, Department of Environmental Immunology, Helmholtz Centre for Environmental Research—UFZ.

Dorret I. Boomsma, Department of Biological Psychology, VU Amsterdam, 1081 BT, Amsterdam, Netherlands.

René Pool, Department of Biological Psychology, VU Amsterdam, 1081 BT, Amsterdam, Netherlands.

Euan Woodward, The European Association for the Study of Obesity, D02 N820, Dublin, Ireland.

Lisa Heggie, The European Association for the Study of Obesity, D02 N820, Dublin, Ireland.

Sheree Bryant, The European Association for the Study of Obesity, D02 N820, Dublin, Ireland.

Rodessa May Marquez, Department of Innovation and Digitalisation in Law, University of Vienna, 1010 Vienna, Austria.

Olga Startseva, Department of Innovation and Digitalisation in Law, University of Vienna, 1010 Vienna, Austria.

Nikolaus Forgó, Department of Innovation and Digitalisation in Law, University of Vienna, 1010 Vienna, Austria.

Eran Segal, Department of Computer Science and Appliance and Applied Mathematics, Weizmann Institute of Science, 7610001 Rehovot, Israel.

Adina Weinberger, Department of Computer Science and Appliance and Applied Mathematics, Weizmann Institute of Science, 7610001 Rehovot, Israel.

Vera Stavroulaki, Wings ICT Solutions Information & Communication Technologies S.A., 17121 Athens, Greece.

Vangelis Argoudelis, Wings ICT Solutions Information & Communication Technologies S.A., 17121 Athens, Greece.

Panagiotis Demestichas, Wings ICT Solutions Information & Communication Technologies S.A., 17121 Athens, Greece.

Danai Malti, Wings ICT Solutions Information & Communication Technologies S.A., 17121 Athens, Greece.

Gianna Karanasiou, Wings ICT Solutions Information & Communication Technologies S.A., 17121 Athens, Greece.

Dimitris Plakas, Wings ICT Solutions Information & Communication Technologies S.A., 17121 Athens, Greece.

Nikos Sintoris, Wings ICT Solutions Information & Communication Technologies S.A., 17121 Athens, Greece.

Vassilios Fanos, Department of Surgical Sciences, University of Cagliari, 09042 Monserrato, Italy.

Angelica Dessì, Department of Surgical Sciences, University of Cagliari, 09042 Monserrato, Italy.

Luigi Atzori, Department of Biomedical Sciences, University of Cagliari, 09042 Monserrato, Italy.

Cristina Piras, Department of Biomedical Sciences, University of Cagliari, 09042 Monserrato, Italy.

Antonio Noto, Department of Biomedical Sciences, University of Cagliari, 09042 Monserrato, Italy.

Patrizia Baire, Department of Surgical Sciences, University of Cagliari, 09042 Monserrato, Italy.

Matteo Mauri, Department of Surgical Sciences, University of Cagliari, 09042 Monserrato, Italy.

Karolina Krystyna Kopeć, Department of Surgical Sciences, University of Cagliari, 09042 Monserrato, Italy.

Cristinel Gheorghiu, BIOCLINICA SA, 300358, Timisoara, Romania.

Anastasios Delopoulos, Department of Electrical and Computer Engineering, Aristotle University of Thessaloniki, 54636 Thessaloniki, Greece.

Ioannis Sarafis, Department of Electrical and Computer Engineering, Aristotle University of Thessaloniki, 54636 Thessaloniki, Greece.

Alexandros Papadopoulos, Department of Electrical and Computer Engineering, Aristotle University of Thessaloniki, 54636 Thessaloniki, Greece.

Chrysa Episkopou, Department of Electrical and Computer Engineering, Aristotle University of Thessaloniki, 54636 Thessaloniki, Greece.

Dimitrios Aletras, Department of Electrical and Computer Engineering, Aristotle University of Thessaloniki, 54636 Thessaloniki, Greece.

Terho Lehtimäki, Department of Clinical Chemistry, Tampere University Hospital and Tampere University, 33520 Tampere, Finland.

Nina Mononen, Department of Clinical Chemistry, Tampere University Hospital and Tampere University, 33520 Tampere, Finland.

Pashupati P Mishra, Department of Clinical Chemistry, Tampere University Hospital and Tampere University, 33520 Tampere, Finland.

Binisha H Mishra, Department of Clinical Chemistry, Tampere University Hospital and Tampere University, 33520 Tampere, Finland.

Leo‐Pekka Lyytikäinen, Department of Clinical Chemistry, Tampere University Hospital and Tampere University, 33520 Tampere, Finland.

Stavroula Mougiakakou, ARTORG Center for Biomedical Engineering Research/AI in Health and Nutrition, University of Bern, 3008 Bern, Switzerland.

Ioannis Papathanail, ARTORG Center for Biomedical Engineering Research/AI in Health and Nutrition, University of Bern, 3008 Bern, Switzerland.

Rooholla Poursoleymani, ARTORG Center for Biomedical Engineering Research/AI in Health and Nutrition, University of Bern, 3008 Bern, Switzerland.

Lubnaa Abdur Rahman, ARTORG Center for Biomedical Engineering Research/AI in Health and Nutrition, University of Bern, 3008 Bern, Switzerland.

Nick Martin, Department of Genetics and Computational Biology, QIMR Berghofer Medical Research Institute, 4029 Brisbane, Australia.

Scott Gordon, Department of Genetics and Computational Biology, QIMR Berghofer Medical Research Institute, 4029 Brisbane, Australia.

Gillian Santorelli, Bradford Institute for Health Research, Bradford Teaching Hospitals NHS Foundation Trust, BD9 6RJ Bradford, United Kingdom.

Ellena Badrick, Bradford Institute for Health Research, Bradford Teaching Hospitals NHS Foundation Trust, BD9 6RJ Bradford, United Kingdom.

John Wright, Bradford Institute for Health Research, Bradford Teaching Hospitals NHS Foundation Trust, BD9 6RJ Bradford, United Kingdom.

Amy Hough, Bradford Institute for Health Research, Bradford Teaching Hospitals NHS Foundation Trust, BD9 6RJ, Bradford, United Kingdom.

Eirini Marouli, William Harvey Research Institute, Queen Mary University of London, EC1M 6BQ London, United Kingdom.

## Data Availability

Data sharing not applicable to this article as no datasets were generated or analysed during the current study.

## Appendix A BETTER4U Partners

The project comprises of 28 partners from multidisciplinary scientific fields, namely: Harokopio University of Athens ‐ HUA (coordinator, WP1 and WP7 leader, data contributor, technical support partner and handler and pilot and RCT clinical site), Aristotle University of Thessaloniki (technical support partner), Centro de Estudos e Investigacao em Dinamicas Sociais e Saudae Associacao sem fins Lucrativos – CEIDSS (data contributor and pilot and RCT clinical site), Universite Claude Bernard Lyon 1‐ UCBL and affiliated entity Hospices Civils Lyon – HCL (pilot and RCT clinical site, Region Hovenstaden ‐ REGIONH (data contributor), European Food Information Council – EUFIC (WP9 leader), International Hellenic University – IHU (data contributor), Karolinska Institutet (pilot and RCT clinical site), Medical University of Vienna ‐ MUW (data contributor), University of Cyprus ‐ UCY (data contributor and pilot and RCT clinical site), University of Helsinki – UH (data contributor), University of Navarra (data contributor and pilot and RCT clinical site), University of Ghent (WP8 leader), SWPS University of Social Sciences and Humanities (WP4 leader, data contributor and pilot and RCT clinical site), University of ‐ UTARTU (WP3 leader and data contributor), Helmholtz Center Munich ‐ German Research Center for Environmental Health – HMGU (data contributor, analyzing site), Vrije Universiteit Amsterdam – VUA (data contributor), Tampere University – TAU (data contributor), the European Association for the Study of Obesity ‐ EASO (dissemination partner), University of Vienna ‐ UNIVIE (WP2 leader), Weizmann Institute of Science – WEIZMANN (data contributor and analyzing site), WINGS ICT Solutions Technologies (technical support partner), University of Cagliari UNICA – analyzing site, BIOCLINICA SA (analyzing partner) and associated partners Born in Bradford ‐ BiB (data contributor), University of Bern ‐ UBERN (WP5 leader and technical support partner), QIMR Berghofer Medical Research Institute ‐ QIMR (data contributor), Queen Mary University of London ‐ QMUL (data contributor).
